# Movements of the Soft-Solids and Liquid of the Blood

**Published:** 1868-01

**Authors:** Rufus King Browne


DENTAL REGISTER.

VOL. XXII.] JANUARY, 1868. [No. 1.

### Original Communications.

SURGEON GENERAL'S OFFICE LIBRARY 22371

### MOVEMENTS OF THE SOFT-SOLIDS AND LIQUID OF THE BLOOD.

BY PROF. RUFUS KING BROWNE, M. D.

THE true test of our opinions upon scientific as well as other problems, is to be found in the contradictions identified with them; for whenever or wherever these contradictions, either implied or involved in our opinions exist, we may be sure that we have not yet discerned the truth in the case.

Since the discovery of the circulation by Harvey, not only has the most marked uncertainty existed as to the true character of the particulars incident to the movement of the blood, but the most obvious confusion, inaccuracy and contradiction exists in the accounts given of the supposed facts.

Sometimes, for instance, a "still layer" (of plasma) is represented as a *fact*. Sometimes this representation of a "still layer" is acceded to or quoted as if the author used the idea for the necessary purpose of description, and yet as if its existence were uncertain and doubtful. Sometimes it is represented that this "still layer," which this time is an undoubted fact, is accounted for by capillary attraction which keeps it stationary. Sometimes (a portion of) the liquid,—excluding note of globules—is represented as moving in the central portion, while again it is said, the plasma occupies the position "*next the walls of the vessel.*" Sometimes it is represented that the corpuscles are "floating in a plasma, which forms a *distinct layer* next the walls of the vessel, which indirectly states the idea that there is no plasma in the axial portion of the tube, and no corpuscles there. Sometimes it is stated that "in vessels of considerable size, as well as the capillaries, the corpuscles occupying the central portion" move with much greater rapidity than the *rest* of the blood, leaving a layer of clear plasma at the sides which is *nearly* (?) immovable. And sometimes, as in the preceding sentence, this sometimes called "still layer" is called "nearly" immovable or stationary.

In presence of facts of life, of the character of which any close observer or student may assure himself, are we not called upon to acknowledge that, we need to ascertain what the truths of the movements of the circulating substances are. Many of those who write upon the physiology of the circulation, appear to have never really given the subject thought enough to avoid these contradictions, while others ignore these palpable defects in a knowledge of the most important of transactions in the organism, under the impression that they by silence conceal their ignorance.

But how is it possible for us to get even a glimpse of the true nature of many morbid processes which we instinctively suppose to have their origin in perturbations, derangement or disturbance of the movement of the blood, without first ascertaining what the character of the movement is, whether it is the same for the different constituents of the blood, or whether one *portion* of the blood, irrespective of its constituents in a certain line in the vessel flows, while another portion on either side of this line, is stationary.

No anatomical or physiological inquiry can possibly be of greater interest, and none can have a more direct and positive bearing upon the condition of many diseases.

The existence of a "still layer" of plasma, is supposed by some to be a fact of *observation*, while others are shown in the preceding contradictions, by alternately ignoring or doubting, and citing it as a fact, not to claim for it that character. Some persons believe, that as there is no *visible movement* of this layer, that this absence of a "sight" of movement, constitutes a fact of observation that there is no movement. But, a "fact of observation" is invariably and always, what we see, *not* what *we do not see.* This latter, is precisely what is *not* a "fact of observation."

In reality, this thin layer of plasma is not a fact, but an *error* of observation. All the plasma in the blood channels moves. It is well known to microscopists, that in the case of a fluid like that of the liquid of the blood, the flow or movement could not be an object of eyesight unless the liquid contained moving particles or bodies. It is their change of position or transit that we *see*, and from that *infer* the movement of the fluid they are in.

Hence, it is only by means of the soft-solid bodies in the blood, that we are enabled to recognise the movement or flow of an otherwise homogeneous fluid. Hence, also, deprive the plasma throughout the calibre of the vessels of these moving bodies, or their equivalent, and we should not be able by *sight* to recognize its flow at all. It is only where such bodies are shifting from point to point, backward (or forward) in the field, that we are enabled to see or visually recognize their motion.

What therefore we recognize in the case of the tide of the blood, is the movement of the corpuscular elements. And hence, in the tide portions, within which there are no such bodies as where the "still layer" occupies, we see no motion, *i. e.* no moving bodies.

Remove the corpuscles from the central portion of the vessel as well as from their sides, and precisely the apparent absence of flow throughout the whole clear volume of plasma would exist. In such a case, *whether* it really was moving so far as the observation of sight was concerned, could only be ascertained by placing in the fluid particles of matter. If these, in themselves inert, were carried along as in a tide, we would at once infer the fluid to be moving.

As therefore, in the instance of this supposed "still layer" we cannot *see the fluid distinct* from the globules move, our conviction that it does must be an apprehension or inference, not an event of eyesight.

This error of observation is, therefore, easily explained by the aid of the foregoing facts. As we saw the globules in a different portion of the vessel from the plasma, move, and did not see the latter, which contained no globules, move, we have named it the "still" or "immovable" layer, or "nearly immovable layer."

In fact, this portion of the plasma at the sides, the so-called "still layer" does move continuously, though its movement, when it alone occupies the sides of the capillary canals, does not coincide with the movement of the plasma, occupying with the globules and between them, the central part of the vessel, for in this portion of the vessel its movement is modified or much hastened, by the rush of the train or column of red globules; and compared with this the flow of the clear plasma is slack.

We had never suspected that the soft-solid bodies of the blood moved, except as moved or rather *carried* by the fluid itself, impelled by the heart; yet noticed again and again the fact, that the white corpuscles moved in this layer, and still we call it immovable.

The fact abundantly *disproves* our theory of such an arrested or stationary part of the blood, which we speak of as a distinct layer, therein alledging a definite line of separation or demarkation between the blood fluid which occupies the centre, and that of the same fluid which adjoins the sides of its channels, designating the plasma according as it was in the centre or elsewhere of the vessel, as two physiologically if not anatomically separate bodies of fluid, one moving through the other, which is stationary.

But far from bearing all the considerations which confront us in holding our present uncertain view, there are others as positively *disproving* of that theory.

According to the theory, that the "still layer" is produced by capillary attraction or power, it exists throughout the capillary system, but in fact, there are various situations in which no clear plasma is situated in the vessels. This is very notably the fact in the entire capillary area of the lung tissue, where the whole calibre of the vessels is invariably found to be filled with red globules. In reality too, the capillaries of nearly every district of the organism, are at any moment and at all times, liable to this filling up of the vessels by red globules, to the displacement of the clear plasma. This is at any time a part of the normal variations or changes in the circulatory apparatus. It is precisely what takes place in those areæ of the capillaries, where periodically congestions occur, as in the capillaries of the stomach in the act of digestion or during the presence of food in the stomach.

But we need hardly dwell any longer upon the erroneous nature of our present views of the circulation, nor augment the number of the facts which bear directly against them. The variations above named, however, are neither according to the theory of "capillary attraction" or "power," nor in fact, can be due to any displacing power of the plasma, for the theory especially *precludes* the moving away or disappearance of the plasma or "still layer," and accounts for its being *stationary*, while the facts are, that it is not stationary, but will be *moved* away or carried onward where there is a sufficient or proportionate number of globules to occupy the space themselves.

But this in which we see the fact that the red globules are not merely *carried* by the plasma, but can move to any part of their circuit at a much quicker rate, and aggregate there in sufficient number to check or even arrest their own movement, and dam the progress of the plasma, is not merely true of the capillaries of certain parts, or other parts at other times, but it may be true of the entire venous system, including the blood vessels to the lungs.

This is the actual physiological condition, (not the disease itself; we must not mistake the *condition* of a thing, for the thing itself,) of the cold stage of fever and ague. If the skin of the arm or chest of a patient in this state be rubbed down by a crash or turkish towel, until the capillaries are denuded and themselves ruptured and broken open, not a drop of *red* blood appears. What is the physiological explanation of this phenomenon? The only possible one is that the red globules, in virtue of their own power of performing the round partly or wholly of the circuit at a faster rate than the fluid, have instead of being distributed throughout the plasma by identity of movement with it, as in normal conditions, are hastened onward and come together nearly entirely in a particular part of the circuit, viz., the small veins, and this disproportion continues there as long as the *condition* of the chill remains. All these various contradictions may be found in nearly the order we have stated, in the following, from the latest compilations on physiology:

"In studying the circulation under the microscope, the anatomical division of the blood into corpuscles and a clear plasma is observed, the corpuscles being comparatively large and floating in a plasma which forms a distinct layer next the walls of the vessels."

"A few white corpuscles were constantly seen in this layer, they move along slowly, and apparently have a tendency to adhere to the walls of the vessels."

"In vessels of considerable size as well as the capillaries, the corpuscles occupying the central portion, move with much greater rapidity than the *rest of the blood*, leaving a layer of clear plasma at the sides which is nearly immovable."

"This curious phenomenon is in obedience to a physical law, regulating the passage of liquids through capillary tubes."

"When we come to the smallest arteries and veins, and still more the capillaries, the capillary attraction is sufficient to produce the *immovable* layer *called* (?) the 'still layer' by many physiologists, and the liquid only moves in the central portion."

"It has been observed that the larger vessels are crowded to their utmost capacity with corpuscles, leaving no *thin layer* next the walls, such as is seen in other situations." (*Flint's Human Physiology*, pp. 228–9.) In exact and unflinching contradiction, Draper cites "capillary power or attraction," and "affinity" to account for the *circulation*, while the citer of Ponssielle cites it to account for the keeping the plasma in a stationary or "still layer," a fixed position in the midst of which runs a rapid current. The first named philosopher appears to imagine that he was the first to apply the idea of capillary power to account for the circulation, and hence he always cites it, and probably will do so for the next fifty years.

The phenomenon described as follows, is due entirely to the afflux and efflux of the red globules, not to the oscillation of the whole blood: "When the circulation has been for a long time under observation," * * * the central organ becomes more and more enfeebled. *At this time* the corpuscles cease to occupy exclusively the central portion of the vessels, and the clear layer of plasma next their walls which was observed in the normal circulation, is no longer apparent." There is actual oscillation in the capillaries. This is explained in the following way: "as the heart has become enfeebled, the contractions are so infrequent and ineffectual, that during their intervals the constant flow in the *capillaries* is entirely arrested. But the feeble impulse of the heart is propagated through the vessels, and *produces a slight impulse* even in the smallest capillaries." This explanation is in direct contradiction to the account of capillary circulation given on page preceding, which reads, "the movement in the capillaries is always quite slow compared with the movement in the arterials, and is continuous. Here at *last the impulse of the heart is lost.*"

The impulse of the heart in health or when pulsating most vigorously is lost, but when so enfeebled as to be near stopping, it is propagated through the vessels, and produces an impulse " even in *the smallest capillaries.*"

Such statements indicate the extremely unsatisfactory state of the learning pertaining to this subject. Also, these contradictions occur in a few paragraphs of a large work.

